# Synthesis and Analysis of Novel Hyaluronic Acid-Based Dual Photocrosslinkable Tissue Adhesive: An In Vitro Study

**DOI:** 10.7759/cureus.58664

**Published:** 2024-04-20

**Authors:** Johnisha Harris, Arvina Rajasekar

**Affiliations:** 1 Periodontics, Saveetha Dental College and Hospitals, Saveetha Institute of Medical and Technical Sciences, Saveetha University, Chennai, IND

**Keywords:** fibrin glue, tissue adhesive, photocrosslinkable, hyaluronic acid, biomaterial

## Abstract

Background

Tissue adhesives are mainly used for aiding in the attachment of adjacent tissues or to nearby hard tissue surfaces. They promote the natural healing processes of the tissues, especially for less painful closure, simple application, no need for sutures following surgery, and localized drug release. This study aimed to synthesize and assess the properties of hyaluronic acid (HA)-based, dual photocrosslinkable tissue adhesive.

Materials and methodology

N-hydroxysuccinimide (NHS), 1-ethyl-3-(3-dimethylaminopropyl)-carbodiimide (EDC), HA, and polymethylmethacrylate, which served as a photoinitiator, were combined to synthesize a tissue adhesive. The prepared formulation was characterized, and its biocompatibility was assessed.

Results

Surface morphology, mechanical properties, and biological properties of the HA adhesive were comparable to those of conventional fibrin glue. Scanning electron microscopy (SEM) analysis showed the average size of the molecules, 10-25 mm in diameter, and also showed a smooth and nonporous surface. The specimens experienced maximum compressive stress of 0.06 ± 0.02 MPa, compressive strain of 3.07 ± 2.02, and a compressive displacement at break of 3.04 ± 1.23 mm, with a maximum force of 2.33 ± 0.07 N at break. The cytotoxicity assay results for HA and fibrin glue are almost equal.

Conclusion

HA-based photocrosslinkable tissue adhesive could be a potential biomaterial in various applications in the field of medicine, especially in soft tissue management.

## Introduction

Tissue adhesives are one of the most rapidly developing medical advances in recent times. It is one of the medical innovations that has developed most quickly in recent years [1]. Tissue adhesive, also known as surgical adhesive or medical glue, is a type of adhesive used in medicine and surgery to close wounds, incisions, or lacerations. It is designed to bond biological tissues together and promote healing without the need for traditional sutures or staples [2-5]. Tissue adhesives are typically made from biocompatible materials that are safe for internal use and can be absorbed or broken down by the body over time. The most common types of tissue adhesives include cyanoacrylates, fibrin sealants, and collagen-based adhesives [6].

Cyanoacrylate adhesives, often referred to as "superglues," are widely used in medical applications. They polymerize quickly in the presence of moisture and form a strong bond between tissue surfaces. These adhesives are often used for closing small superficial wounds, such as lacerations on the skin, but are known to have an exothermic reaction, which might harm the tissues [7]. Fibrin sealants are composed of fibrinogen and thrombin, two proteins involved in the blood clotting process. When combined, these proteins form a clot-like substance that adheres to the wound, sealing it and promoting healing. Fibrin sealants are often used in surgeries involving blood vessels or other delicate tissues [8].

Collagen-based adhesives are derived from animal sources and contain collagen proteins. These adhesives mimic the natural extracellular matrix found in tissues and promote cell migration and tissue regeneration. They are commonly used in ophthalmic and neurological surgeries [9]. Tissue adhesives have several advantages over traditional sutures or staples. They can be applied quickly, reducing procedure time and potentially leading to improved patient comfort. Adhesives also provide a barrier against bacteria, reducing the risk of infection. Additionally, they eliminate the need for suture removal, as most tissue adhesives are absorbed or sloughed off naturally as the wound heals [10].

Hydrogels are another type of tissue adhesive that consists of water-absorbing polymers. These adhesives are biocompatible and can adhere to wet surfaces, making them suitable for use in moist environments, such as the gastrointestinal tract or mucosal surfaces. Hydrogels are used by various medical fraternities for wound closure, drug delivery systems, and tissue engineering [11]. Hyaluronic acid (HA) is a naturally found compound present in the human body in connective tissues and fluids such as synovial fluid. It is a glycosaminoglycan, which means it is a long chain of repeating sugar molecules. HA has also gained limelight in the field of medicine and cosmetics due to its unique properties, including its ability to retain water and provide lubrication [12].

The goal of this study was to develop a dual photocrosslinkable HA-based tissue adhesive, analyze its mechanism, describe its unique properties, discuss the benefits and drawbacks of using it in clinical settings, and find ideas for future research projects aimed at creating the next generation of tissue adhesives.

## Materials and methods

Preparation of adhesive

Initially, 10× phosphate-buffered saline (PBS) was formulated by adding 10 mL of PBS with 90 mL of distilled water, which was later mixed with HA. In a separate container, 120 mg of N-hydroxysuccinimide (NHS) was added to 55 mg of 1-ethyl-3-(3-dimethylaminopropyl)-carbodiimide (EDC). The latter was mixed with activated HA. This product was kept at -80°C to ensure that the residue became sufficiently sedimented to be separated on its own. After that, ethanol was used to wash the residue four or five times. The supernatant solution was removed. Afterward, the pellets were stored at -80°C. Then, the moisture was extracted by vacuum-drying. Next, a 300-mL beaker containing 0.1 L of deionized water and 1 g of HA was combined. After that, 50 mL of deionized water was mixed with 10 g of sodium hydroxide in a different beaker. This was followed by the transfer of 2 mL of NaOH to a 100 mL beaker. Finally, 7.8 mL of poly(methyl methacrylate), a photoinitiator, was added to this mixture.

Characterization

Visual Observation

Scanning electron microscopy (SEM) analysis was done to analyze the structural characteristics of the prepared adhesive sample (Japan Electron Optics Laboratory (JEOL) JSM-IT800, JEOL (Germany) GmbH, Freising, Germany, platinum sputter). The adhesive samples were split into multiple pieces using a razor blade. Before SEM analysis, the specimen was sputtered by platinum in a vacuum (JEOL, JFC-1600, Tokyo, Japan). The glue was characterized by Fourier-transform infrared spectroscopy (FTIR) with the aid of attenuated total reflectance (ATR) mode. The wave number ranged from 500 to 5,000 cm^-1^ at a resolution of 1 cm^-1^, with an average of 64 scans. The equipment used was Bruker Alpha II - compact Fourier transform infrared spectrometer with platinum ATR. In a 1:5 ratio, freeze-dried adhesive was powdered and mixed with potassium bromide.

Strength Test

**Figure 1:**
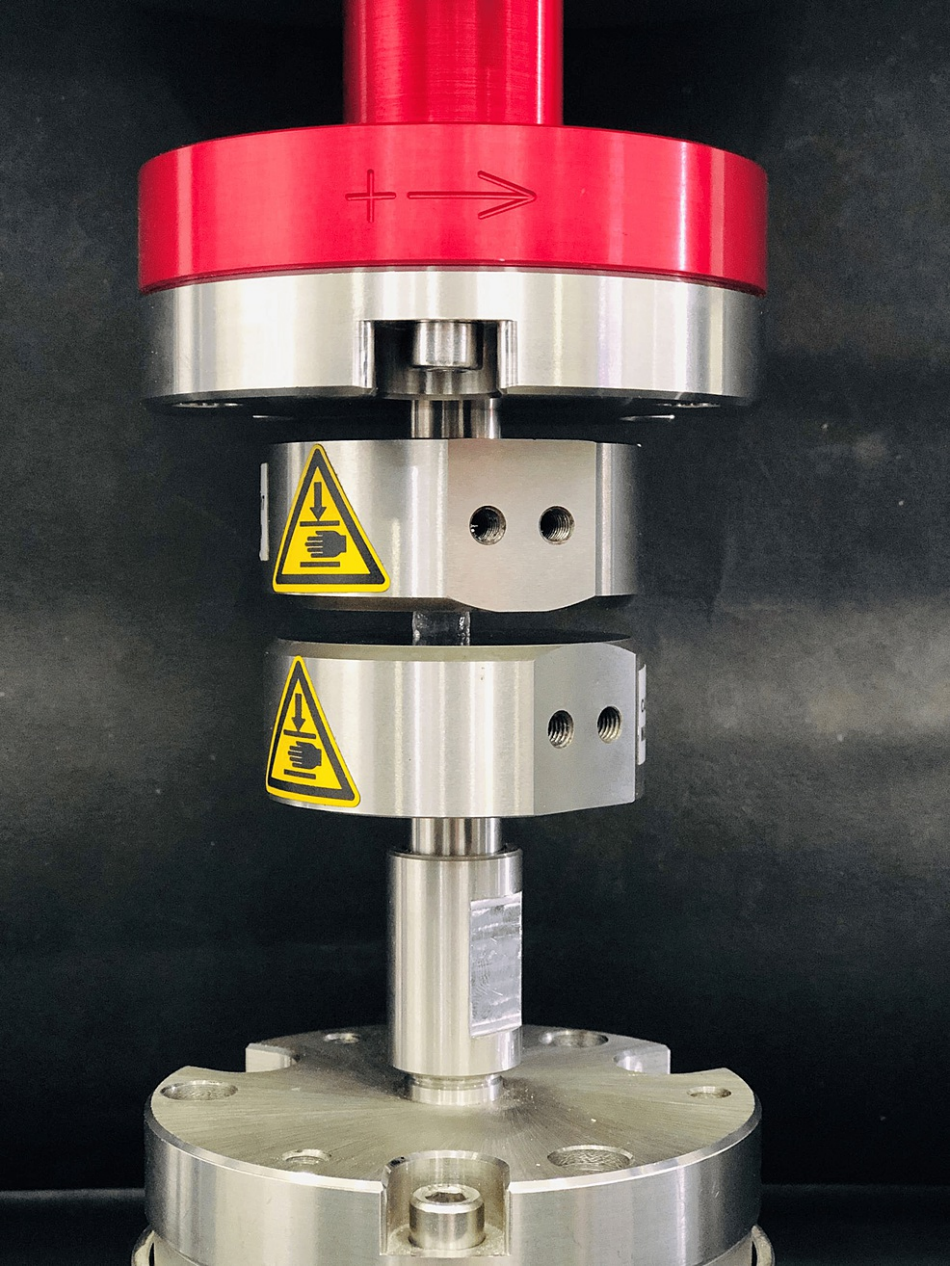
Compressive strength analysis using a universal testing machine

Compressive strength was evaluated with the help of a dynamic universal testing machine (UTM) (Instron Electroplus E3000), as shown in Figure 1. Specimen dimensions were 150 × 10 mm^2^ and a distance of 100 mm between the grips initially. This was in accordance with the American Society for Testing Materials (ASTM) standard. One end of the specimen (fixed) was retained in the upper cross-head of the machine. The specimen was gradually loaded by the machine's loading unit while its other end was attached to the movable, adjustable cross-head.

Cell Viability Analysis

A LIVE/DEAD fluorescence assay kit (produced by Molecular Probes) (Sigma Aldrich, Burlington, USA) was used to assess the viability test. In a qualitative biocompatibility test, Living Dead® (Sigma Aldrich, Burlington, USA) is utilized. The cells were seeded (3 × 106 cells/mL) in a 96-well plate and cultivated for 24 hours at 37°C in Dulbecco’s Modified Eagle Medium - Low Glucose (DMEM-LG) with 10% FBS. The cells were then cultured for 24 hours using the poly-l-lactic acid (PLLA) scaffolds, which measure 2 mm by 5 mm. Following a 24-hour period, the cells were cleaned with 200 L of PBS and then treated with the calcein acetoxymethyl ester (calcein-AM) and ethidium homodimer-1 solution per the manufacturer's instructions. Following a half-hour incubation at 37°C, the cells were cleaned and stored in PBS. The cells were analyzed using the Nikon E800's inverted fluorescence microscopy program (Image Pro-Plus software, Media Cybernetics, Rockville, USA).

## Results

SEM analysis

**Figure 2:**
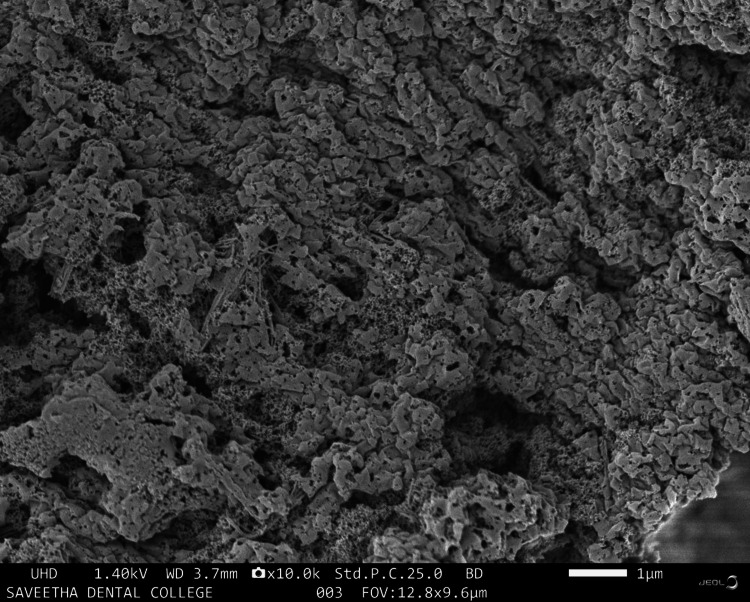
SEM image of HA tissue adhesive BD, beam deceleration; FOV, field of view; HA, hyaluronic acid; SEM, scanning electron microscopy; UHD, ultra high definition; WD, working distance

Microstructural morphological characteristics of the HA-based photocrosslinkable tissue adhesive is shown in Figure 2. SEM analysis verified that the average size of the molecules was 10-25 mm in diameter, showing a smooth and nonporous surface.

FTIR analysis

**Figure 3:**
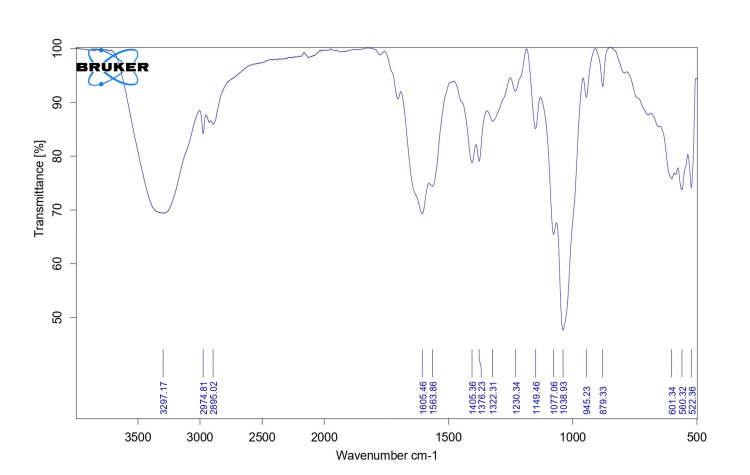
FTIR image of HA tissue adhesive FTIR, Fourier-transform infrared spectroscopy; HA, hyaluronic acid

FTIR analysis reveals the photocross linkage between HA and photoinitiator. The peak wave was seen at 3,297.17 and 1,077.5 cm^-1^, which indicates O-H and C-O structure, respectively (Figure 3).

Compressive strength

**Figure 4:**
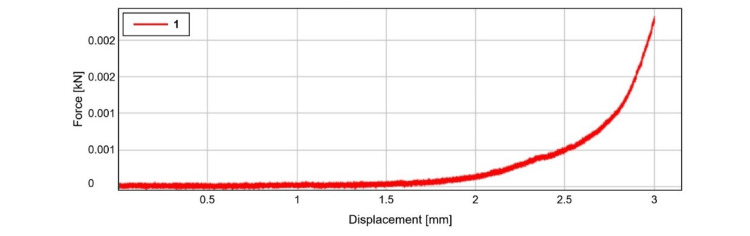
Graphical representation of compressive strength of the specimen

The test specimen's compressive strength was assessed with the aid of the Instron Universal Testing Machine (Instron, Norwood, USA). As seen in Figure 4, the displacement for the specimen was 3 mm at 0 kN. The specimens experienced maximum compressive stress of 0.06 ± 0.02 MPa, compressive strain of 3.07 ± 2.02, and compressive displacement at break of 3.04 ± 1.23 mm at maximum force of 2.33 ± 0.07 N at break. The compressive strength of the specimen was depicted graphically in Figure 4. The average compressive strength analysis values of the samples are tabulated in Table 1.

**Table 1:** Compressive strength analysis of the HA-based tissue adhesive

Maximum force (N)	Compressive displacement at break (standard) (mm)	Compressive strain (displacement) at break (standard) (mm)	Compressive stress at break (standard) (MPa)
2.33 ±0.07	3.04 ± 1.23	3.07 ± 2.02	0.06 ± 0.02

Viability by LIVE/DEAD®

**Figure 5:**
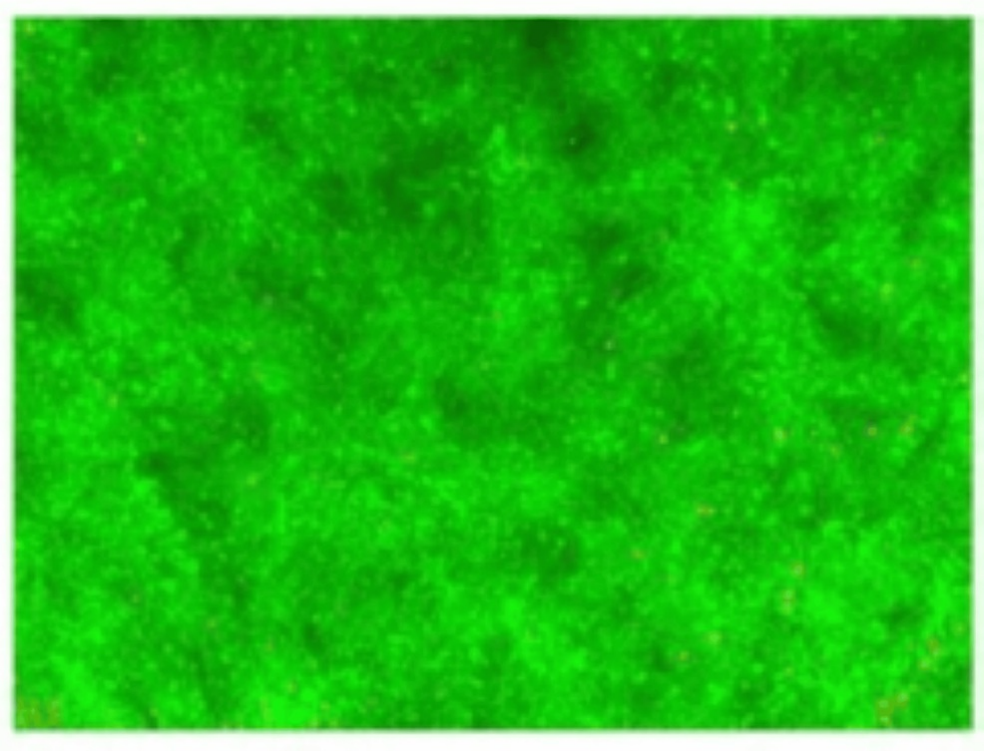
Cell viability of fibrin glue-based tissue adhesive

**Figure 6:**
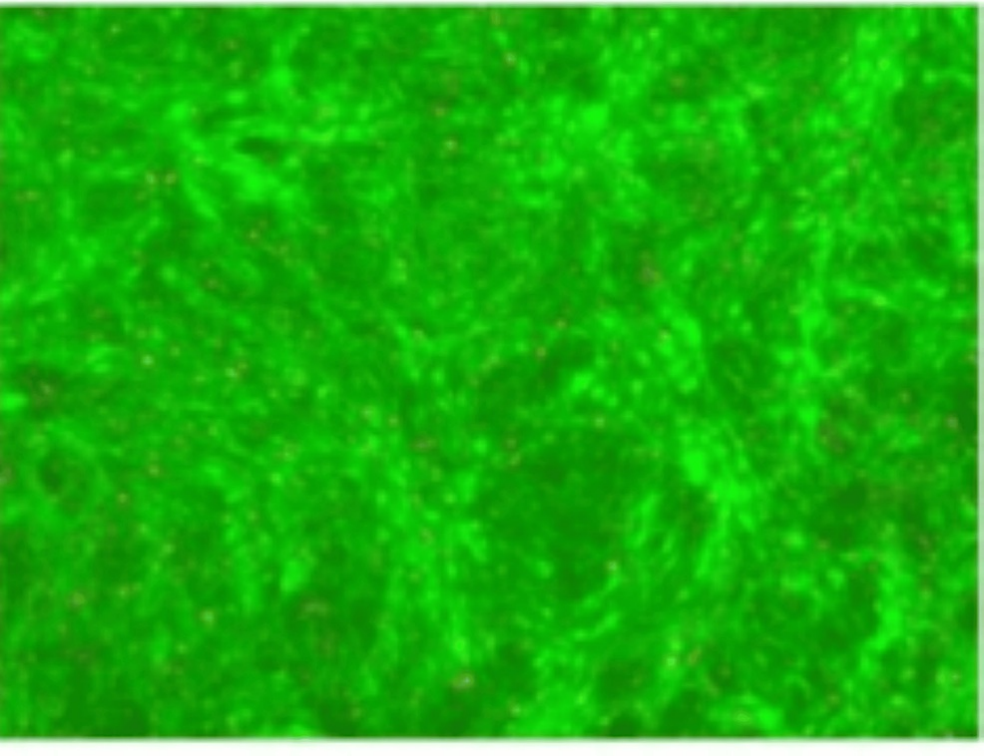
Cell viability of HA tissue adhesive HA, hyaluronic acid

The polymer's biocompatibility with cells is qualitatively demonstrated by the LIVE/DEAD® experiment. The cells were grown using the biomaterial. Living cells interacted with the fluorescent marker SYTO® 9 (Thermo Fisher Scientific Inc., Waltham, USA), which resulted in the green staining of healthy cells. Conversely, unviable cells were dyed red, signifying that they were dead cells. The LIVE/DEAD® technique is illustrated in Figure 5 and Figure 6 using the results of inverted fluorescence microscopy. Our findings from the cytotoxicity test revealed that while the fibrin glue increased the number of visible dead cells, the HA-based tissue adhesive enhanced the growth of L929 cells than the former. This demonstrates the efficacy and biocompatibility of our artificial HA-based tissue adhesive.

## Discussion

Biomaterials, which are used as tissue adhesives, have a crucial role in cell behavior, such as cell adhesion and gene expression, mainly because of their consistency. Thus, maintaining the mechanical properties of the materials would be advantageous [13]. Our study data showed that the stiffness of the HA-polyethylene glycol (PEG) hydrogels may be changed by adjusting the pH of a solution containing PEG-(NH2)6 prior to gelation. Two competing reactions - hydrolysis and reactivity with primary amines - with the NHS-activated carboxyl groups of HA occur simultaneously [14]. Carboxyl groups are produced through hydrolysis, while amide bonds are produced by reaction with primary amines. Typically, both reactions take place more frequently at higher pHs, with amide production increasing the most. Yet as the reaction progresses in this instance, the molecular weight of both polymers rises [15]. This causes the polymers containing carboxyl groups with NHS activation and primary amines to diffuse at slower rates, which lengthens the time it takes for the reaction between two functional groups [16]. Due to the slower diffusion rate, it is more likely that hydrolysis occurs first, before the reaction between two reactive groups. As a result, the reaction kinetics slow down when the pH is lowered, giving primary amines more time to travel to NHS-activated carboxyl groups and engage in reaction to generate amide bonds. As the number of amide bonds increases, the hydrogel becomes more crosslinked. This results in enhanced stiffness, as indicated by the greater compression modulus. This stiffness is managed with the help of altering the pH. When the density of cross-linkage and modulus rises, with the same composition in hydrogels synthesized from the same polymer, the equilibrium swelling ratio decline is predicted. The measured ratio of swelling was reduced when pH declined, as was predicted, which is in line with the compression modulus results. The variable HA content is responsible for these variabilities in the values [17].

Tissue integration has been found to be enhanced in terms of adherence to the underlying tissue. Here, we demonstrate that HA gels have a 10 times higher adherence to cartilage tissue compared to fibrin glue. Apart from creating mechanical crosslinking to the adjacent tissues, similar to fibrin glue, HA-based gel can initiate covalent crosslinks by building amide bonds with the primary amine groups located on collagen in the extracellular matrix [18]. Thus, the increased covalent crosslinks are responsible for the material's improved adhesion properties [19].

These findings are similar to those of previous studies done with HA as a tissue adhesive. Ming Li et al. explored the possibility of a novel HA hydrogel adhesive, which is based on phenylboronic acid-diol ester linkages. HA-3-(acrylamido)phenylboronic acid (HA-3APBA) hydrogels, formed through phenylboronic acid (PBA) and diols complexation, exhibit injectability, suitable mechanical properties, and a functional molecule for tissue binding. In vivo studies confirm the hydrogel's effectiveness in biocompatibility, wound exudate absorption, controlling bleeding, and accelerating wound closure. It outperforms commercial fibrin glue in adhesion tests [20].

Another study by Koivusalo et al. proved that dopamine-modified HA hydrogel enhanced tissue adhesion, enabled protein conjugation, and supported stem cell culture. It improved cell viability and mechanical properties and reduced swelling in cell culture [21]. The study by Milne et al. evaluated HA methacrylate aldehyde dual crosslinked network (HA-MA-CHO-DCN) hydrogels' adhesive performance through lap-shear and burst pressure tests, comparing them to bovine serum albumin glutaraldehyde (BSAG) glue hydrogels commonly used in surgery. The HA-MA-CHO-DCN hydrogels showed greater burst pressure in comparison to the control sealant, meeting requirements for various human tissue applications [22].

HA was combined with dopamine (HA-DN) along with thiol end-capped Pluronic F127 copolymer (Plu-SH), creating an HA/Pluronic composite gel, and the resulting hydrogels showed phase transitions that can vary depending on the temperature, with rapid sol-gel behavior, which is reversible. These hydrogels could be injected as a sol at room temperature and would quickly gel at body temperature, demonstrating excellent tissue adhesion and stability for potential drug and cell delivery applications [23]. An adhesive hydrogel made of MeHA and ELP developed by Shirzaei Sani et al. with antimicrobial properties and elastic behavior can be quickly photocrosslinked for tissue healing. MeHA/ELP hydrogels showed no significant inflammatory response, were effectively biodegradable, and facilitated the integration of new autologous tissue [24].

All the previous studies have discussed the physical and biochemical properties of the HA and the photocrosslinking property, but most of them are used in other specialities and not in dentistry. The results of these study validates the use of HA in tissue adhesives because of favorable compressive strength and biocompatibility like fibrin glue.

Limitations

Although the prepared HA-based tissue adhesive exhibited better mechanical and biological properties, clinical studies are further needed to substantiate these findings. The rate of degradation in the oral environment should also be analyzed in future studies.

## Conclusions

HA-based tissue adhesives are promising candidates for a wide range of procedures especially in soft tissue management. The strength of this bio adhesive is comparable to the commercially available adhesive fibrin glue. Therefore, the HA-based photocrosslinkable adhesive could be a potential biomaterial in various applications in the field of medicine including dentistry for procedures like flap and mucogingival surgeries.
